# Development of Hot-Extruded Mg–RE–Zn Alloy Bar with High Mechanical Properties

**DOI:** 10.3390/ma12101722

**Published:** 2019-05-27

**Authors:** Zehua Li, Jinghuai Zhang, Yan Feng, Jinshu Xie, Yinfu Liu, Shujuan Liu, Jian Meng, Qiang Yang, Zhuang Liu, Ruizhi Wu

**Affiliations:** 1 Key Laboratory of Superlight Material and Surface Technology, Ministry of Education, College of Material Science and Chemical Engineering, Harbin Engineering University, Harbin 150001, China; zehuali163@163.com (Z.L.); fy1102900326@163.com (Y.F.); xjs2018@hrbeu.edu.cn (J.X.); cubeyin98@163.com (Y.L.); rzwu@hrbeu.edu.cn (R.W.); 2 Department of Materials Physics and Chemistry, Harbin Institute of Technology, Harbin 150001, China; liusj0817@hit.edu.cn; 3 State Key Laboratory of Rare Earth Resources Utilization, Changchun Institute of Applied Chemistry, Chinese Academy of Sciences, Changchun 130022, China; jmeng@ciac.ac.cn (J.M.); qiangyang@ciac.ac.cn (Q.Y.); 4 Faculty of Materials Science and Engineering, Kunming University of Science and Technology, Kunming 650093, China; liuzhuang0527@163.com

**Keywords:** Mg alloys, hot extrusion, microstructure, mechanical properties, stacking faults

## Abstract

A new elevated-temperature high-strength Mg–4Er–2Y–3Zn–0.4Mn (wt %) alloy was developed by semi-continuous casting, solid solution treatment, and hot extrusion. W phase (Mg_3_(Er,Y)_2_Zn_3_) with fcc structure, long period stacking ordered phases with 18R (Mg_10_(Er,Y)_1_Zn_1_) and 14H (Mg_12_(Er,Y)_1_Zn_1_) structures, and basal plane stacking faults (SFs) was formed in the as-cast alloy, mainly due to the alloy component of (Er + Y)/Zn = 1:1 and Er/Y = 1:1 (at %). After solid solution treatment and hot extrusion, the novel microstructure feature formed in as-extruded alloy is the high number-density nanospaced basal plane SFs throughout all the dynamically recrystallized (DRXed) and un-DRXed grains, which has not been previously reported. The as-extruded alloy exhibits superior tensile properties from room temperature to 300 °C. The tensile yield strength can be maintained above 250 MPa at 300 °C. The excellent elevated-temperature strength is mainly ascribed to the formation of nanospaced basal plane SFs throughout the whole Mg matrix, fine DRXed grains ~2 μm in size, and strongly basal-textured un-DRXed grains with profuse substructures. The results provide new opportunities for the development of deformed Mg alloys with satisfactory mechanical properties for high-temperature services.

## 1. Introduction

Magnesium (Mg) is one of the most abundant light metal elements on earth [1], and at present, Mg alloys are the lightest structural materials in the practical engineering applications. Mg alloys, known as “the green engineering materials for the 21st century”, have the characteristics of high specific strength, excellent damping, easy recycling, etc., and have therefore attracted more and more attention for their application in lightweight designs in the automotive, aerospace, and electronic industries [2,3,4,5,6,7]. However, common Mg alloys also have their own weaknesses, such as low absolute strength, especially at elevated temperature, which is one of the reasons why Mg alloys have not been widely used [8,9,10]. At present, the popular commercial Mg alloys, such as the AZ, AM, and ZK series, are of room temperature (RT) Mg alloys whose strength is moderate at RT, while deteriorates sharply with increasing temperatures [11]. Therefore, the development of new Mg alloys with excellent mechanical properties, particularly at elevated temperatures, is of great importance [12].

The most effective way to improve the mechanical properties of large-scale Mg alloy materials is to adjust the microstructure via alloy composition design and plastic deformation processing [13]. Rare earth (RE) elements have been considered to be the extremely useful and promising alloying elements for steels or non-ferrous alloys [14,15], especially for heat-resistant Mg alloys [16,17,18,19]. Among all the RE-containing Mg alloys, the Mg–RE–Zn (where RE is a heavy rare earth element such as Y, Gd, Dy, Ho, Er) system has great development potential due to formation of some strengthening phases with special structures based on the RE/Zn ratio [17]. With the increasing RE/Zn ratio, the reinforcing phases with quasicrystal structure [20], cubic structure [21], and long period stacking order (LPSO) structure [22], as well as basal plane stacking faults (SFs) [23,24] have been found in these Mg–RE–Zn alloys. Moreover, previous reports have suggested that these phases/structures should provide superior strengthening effects under the better regulation of morphology, size, number density, and distribution [17]. Hot extrusion, as a typical deformation processing technology of Mg alloys, can not only refine the Mg grain size, but also change the size and distribution of secondary phases, and sometimes also can promote dynamic formation/precipitation based on the reasonable alloy composition and appropriate extrusion process [25,26]. All the above microstructure changes contribute greatly to the strengthening of Mg alloys. At present, some RT high-strength or even ultrahigh-strength Mg–RE–Zn alloys, such as Mg–Y–Zn, Mg–Gd–Zn, and Mg–Gd–Y–Zn, have been fabricated [17,27], but few reports refer to the elevated-temperature mechanical properties for this series of alloys, particularly for the SF-containing Mg–RE–Zn alloys.

Based on our research experience and the foundation of Mg–RE–Zn alloys [28], we prepared, in this work, a new Mg–0.6Er–0.6Y–1.2Zn–0.2Mn (at %) (i.e., Mg–4Er–2Y–3Zn–0.4Mn, wt %) alloy based on (Er + Y)/Zn = 1:1 and Er/Y = 1:1 (at %). The addition of a small amount of Mn is mainly considered to be beneficial to the corrosion resistance [29]. Some novel microscopic characteristics, different from those of conventional deformed Mg alloys, have been found in this new hot-extruded alloy bar. Furthermore, the new hot-extruded alloy bar exhibits excellent mechanical properties, especially at elevated temperatures. The results of this study form a basis for the development of high-temperature high-performance deformed Mg alloys.

## 2. Materials and Methods

The casted rod was prepared by a direct-chill semi-continuous casting method. The Mg–4Er–2Y–3Zn–0.4Mn (wt %) alloy was prepared from highly pure Mg (99.98 wt %), Zn (99.95 wt %), and Mg–20Y (wt %), Mg–20Er (wt %), and Mg–10Mn (wt %) master alloys in an electric resistant furnace under the protection of Ar atmosphere. After stirring and then standing at ~730 °C for 0.5 h, the alloy melt was poured into the cooling crystallizer with a diameter of 100 mm at a casting speed of about 100 mm/min at ~710 °C. The actual chemical composition of this alloy is Mg–4.03Er–1.95Y–3.14Zn–0.34Mn (wt %), i.e., Mg–0.63Er–0.57Y–1.26Zn–0.16Mn (at %), determined by inductively coupled plasma analyzer (ICP, Optima 8000DV, perkinElmer, Waltham, MA, USA), which was quite close to the designed one. The ingot was machined into the cylindrical-shaped billets with a diameter of 80 mm, and then solid solution-treated at 530 °C for 10 h. These homogenized billets were extruded into bars at 350 °C with an extrusion ratio of 9.5 under the extrusion ram speed of 0.3 mm·s^−1^, and subsequently quickly quenched into cold water.

The microstructure was characterized by scanning electron microscope (SEM, Merlin Compact, Carl Zeiss, Jena, Germany) equipped with an X-ray energy-dispersive spectrometer (EDS, Oxford, Oxford Instruments, Oxford, UK), X-ray diffraction (XRD, Bruker D8 Advance, Bruker AXS, Karlsruhe, Germany) with Cu K_α_ radiation, electron backscatter diffraction (EBSD, NordlysMax3, Oxford Instruments), and transmission electron microscope (TEM, Tecnai G20, FEI, Hillsboro, OR, USA). The thin foil samples for TEM observation were prepared using the argon ion thinning technique. The specimens for tensile test were dumbbell test pieces with a gauge size of 46 mm × 8 mm × 2 mm, and their length direction was parallel to the extrusion direction (ED) for the as-extruded alloy. Displacement-controlled tensile testing was performed using an Instron 5869 testing machine having extensometer (Instron, Norwood, MA, USA) with an initial strain rate of 1×10^−3^ s^−1^ at RT and high temperatures. Each high-temperature tensile specimen would be held for 10 min before testing, and the tensile data were an average value of at least four tensile specimens.

## 3. Results and Discussion

### 3.1. Microstructure of As-Cast Ingot

**Figure 1:**
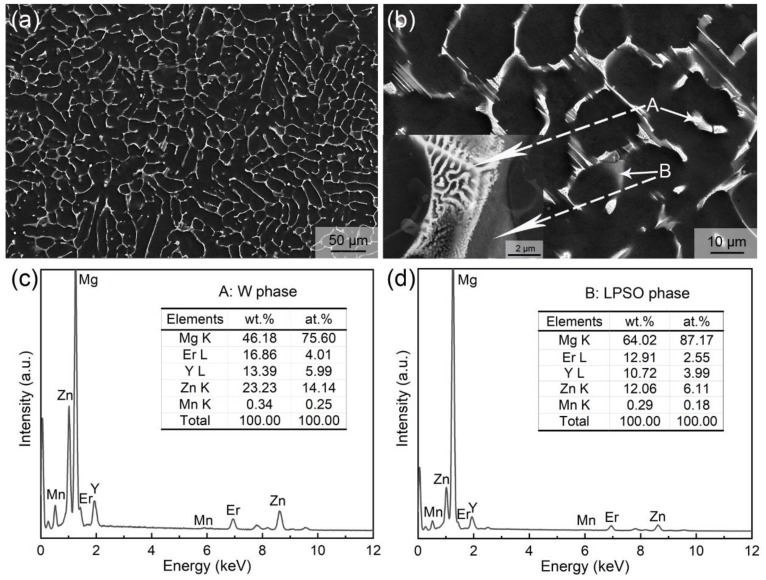
Microstructure of the as-cast alloy: (**a**,**b**) SEM images; EDS results of the phases (**c**) marked as A and (**d**) marked as B in (**b**).

**Figure 2:**
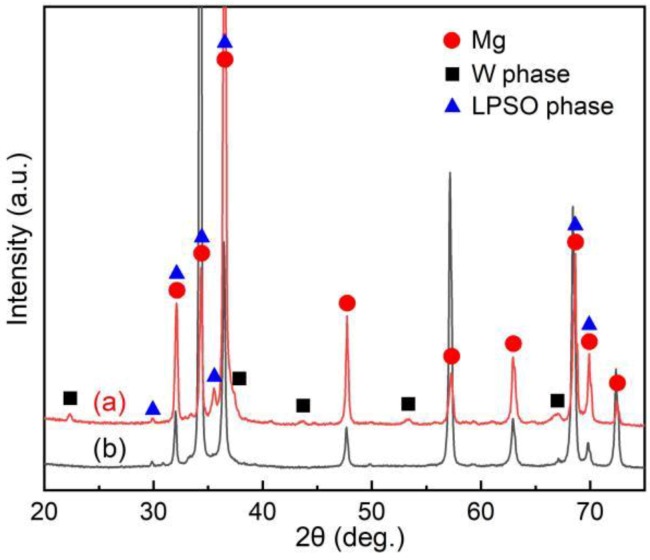
XRD patterns of the (**a**) as-cast and (**b**) as-extruded alloys.

Figure 1 shows the SEM micrographs and corresponding EDS result of as-cast alloy. At low magnification (Figure 1a), a dendritic structure mainly consisting of α-Mg matrix and interdendritic eutectics was observed, which is the typical microstructure of Mg alloys in the as-cast state. The dendritic arm spacing (DAS) is about 30 µm, as determined by the linear intercept method. The volume fraction of eutectics is about 22.2% in total, as shown using an image analysis method. However, under high-magnification observation (Figure 1b), the interdendritic eutectic compounds present two morphologies, one with a typical eutectic structure (marked as A), and the other with a gray divorced eutectic morphology (marked as B). In addition, they are generated alternately with parallel interfaces, and it is related to the alloy composition and the non-equilibrium solidification casting process [30]. EDS results show that the atomic ratio of Mg, (Er + Y), and Zn is close to the chemical composition of W phase (Mg_3_RE_2_Zn_3_) in the typical eutectic phase, while it is close to that of LPSO phases (Mg_12_RE_1_Zn_1_ or Mg_10_RE_1_Zn_1_) in the gray divorced eutectic phase. In SEM–EDS mode, the obviously excess Mg atoms were detected from Mg matrix, due to the small size of the compounds. Figure 2 presents the XRD patterns of the studied alloy in as-cast and as-extruded states. The results suggest that the XRD peaks of as-cast alloy are close to the standard diffraction peaks of Mg, Mg_12_Y_1_Zn_1_, and Mg_3_Y_2_Zn_3_ phases, which agrees with the SEM–EDS result.

**Figure 3:**
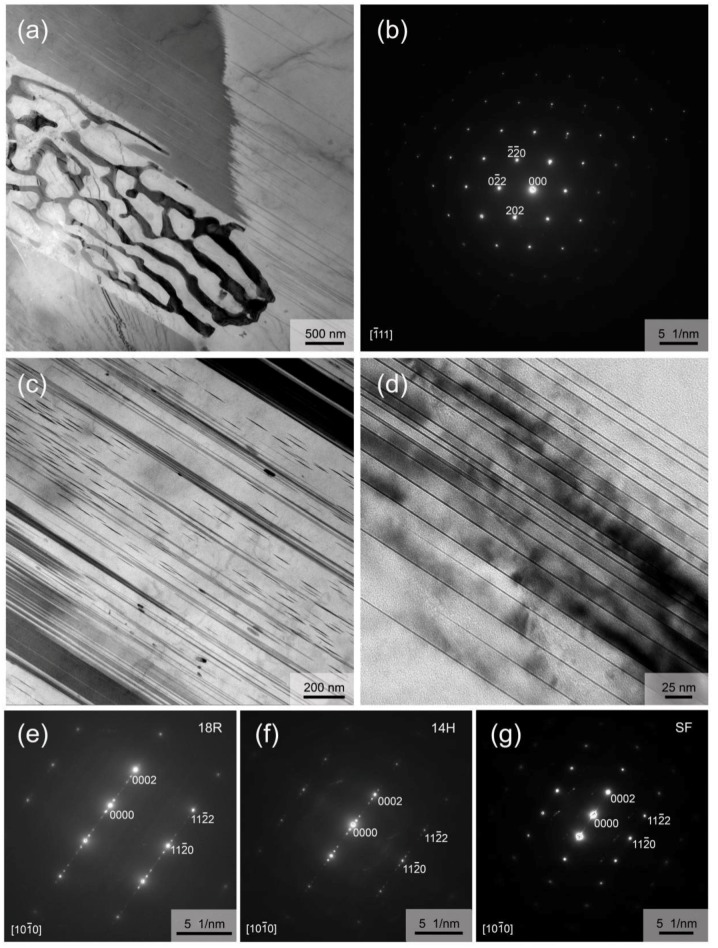
Microstructure of the as-cast alloy: (**a**,**c**,**d**) TEM images; (**b**,**e**–**g**) SAED patterns.

To further confirm the secondary phases formed in as-cast alloy, the phase morphology and structure were characterized by TEM. Figure 3a shows the mixed structure of the typical eutectic phase and plate-like compounds with lamellar structure. The typical eutectic phase is confirmed as the W phase (Mg_3_(Er,Y)_2_Zn_3_) with fcc structure, as determined by the corresponding selected area electron diffraction (SAED) pattern analysis (Figure 3b) combined with SEM–EDS and XRD results. The thickness of the plate-like compounds varies from tens of nanometers to several microns (Figure 3a,c). Figure 3e shows the [10$\bar{1}$0]_Mg_ SAED pattern having extra diffraction spots at ±n/6 (0002)_Mg_ (n is an integer), and Figure 3f shows the [10$\bar{1}$0]_Mg_ SAED pattern having extra diffraction spots at ±n/7 (0002)_Mg_ (n is an integer), which is the evidence commonly used in previous studies to prove the existence of the 18R and 14H structure, respectively [31]. Thus, careful SAED analysis indicates 18R and 14H LPSO structures coexist in the plate-like compounds, which is agreement with the finding in Mg–Y–Zn alloys [31]. Combining our results with the findings of Nie et al. [31], the phase with 18R LPSO structure in this alloy should be Mg_10_(Er,Y)_1_Zn_1_ composition, and the phase with 14H LPSO structure should be Mg_12_(Er,Y)_1_Zn_1_ composition. Furthermore, fine nanoscale lamellae (Figure 3c) and fine lamellae with a large aspect ratio (Figure 3d) are also observed by TEM. The SAED pattern, having streaks between the diffraction spots along the c-axis (Figure 3g) in conjunction with the reported results in the literature [32,33] indicate that they are basal plane stacking faults (SFs).

### 3.2. Microstructure of Solid Solution-Treated Alloys

**Figure 4:**
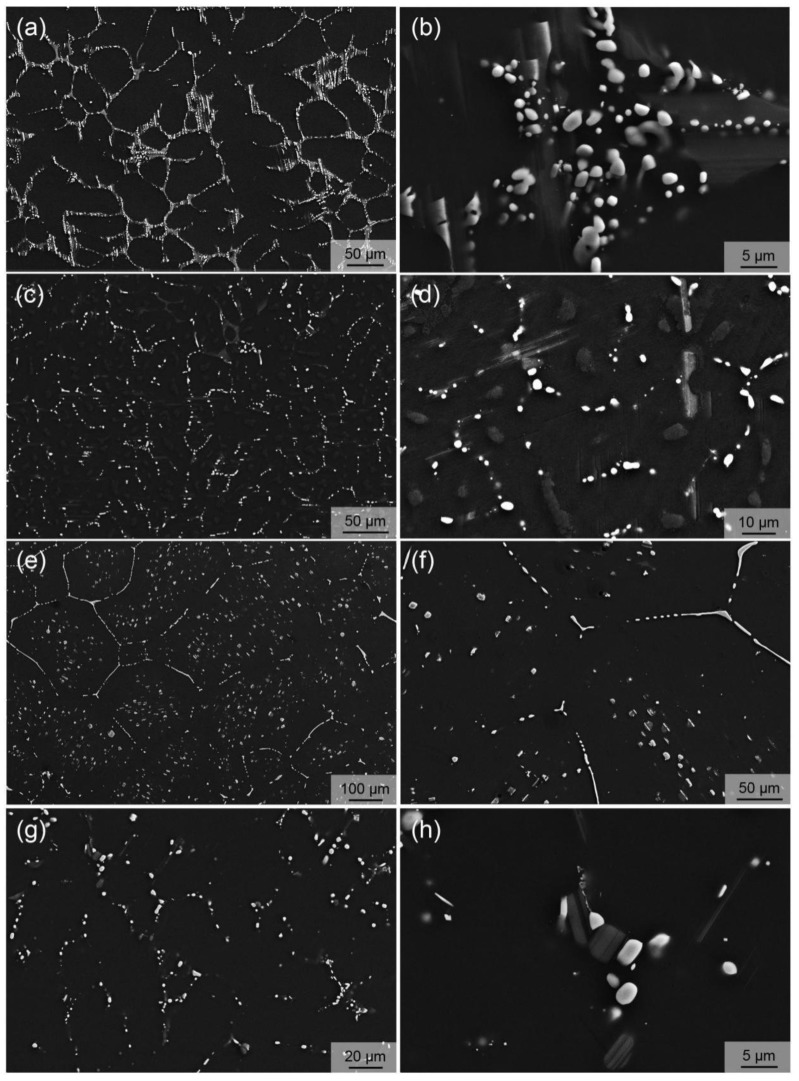
SEM images of the alloys after solid solution treatment at (**a**,**b**) 510 °C for 12 h, (**c**,**d**) 515 °C for 12 h, (**e**,**f**) 530 °C for 12 h, and (**g**,**h**) 530 °C for 10 h.

Figure 4 shows some typical microstructures of solid solution-treated alloys, revealing that the heat treatment temperature and time are very critical for the optimal heat treatment condition. After being heat-treated at 510 °C for 12 h, the microstructure of the alloy seems to change little compared with that of the as-cast alloy observed from the low-magnification SEM micrograph (Figure 4a), that is, the eutectic phases are only slightly dissolved into the Mg matrix, indicating an insufficient solid solution condition, while from the high-magnification SEM image (Figure 4b), the W phase compounds have an obvious spheroidization trend and the LPSO phase has little change, suggesting that LPSO phases has higher thermal stability than the W phase. When the temperature was raised to 515 °C for the 12 h heat treatment (Figure 4c,d), there was a relatively large change in microstructure, but the solid solution condition was still insufficient. Besides spherification, more W phase particles are dissolved into the Mg matrix. In addition, solute atoms apparently tend to segregate within grains, a trend that becomes more pronounced as temperatures continue to rise. Figure 4e,f shows the SEM images of the sample after being heat-treated at 530 °C for 12 h, revealing significant microstructure changes and, also, the unsuitable solid solution condition. A semi-continuous phase is formed along the boundaries of grains, obviously growing up, and moreover, a large number of spheroid-like particles with a size of ~5 µm appear within grains. Finally, we found the superior solid solution condition to be 530 °C for 10 h, as shown in Figure 4g,h. By comparing Figure 1a,b with Figure 4g,h, it can be seen that most of the secondary phases were solidly dissolved into the Mg matrix, and only about 3.5% of the secondary phases remain. The total content of eutectic phases was reduced by about 84.2% as compared to the as-cast alloy. In addition, the grain size of solid solution-treated alloy seemed to only change slightly compared with that of the corresponding as-cast alloy. Therefore, 530 °C for 10 h was selected as the solid solution treatment condition to be used before hot extrusion.

### 3.3. Microstructure of As-Extruded Alloy

**Figure 5:**
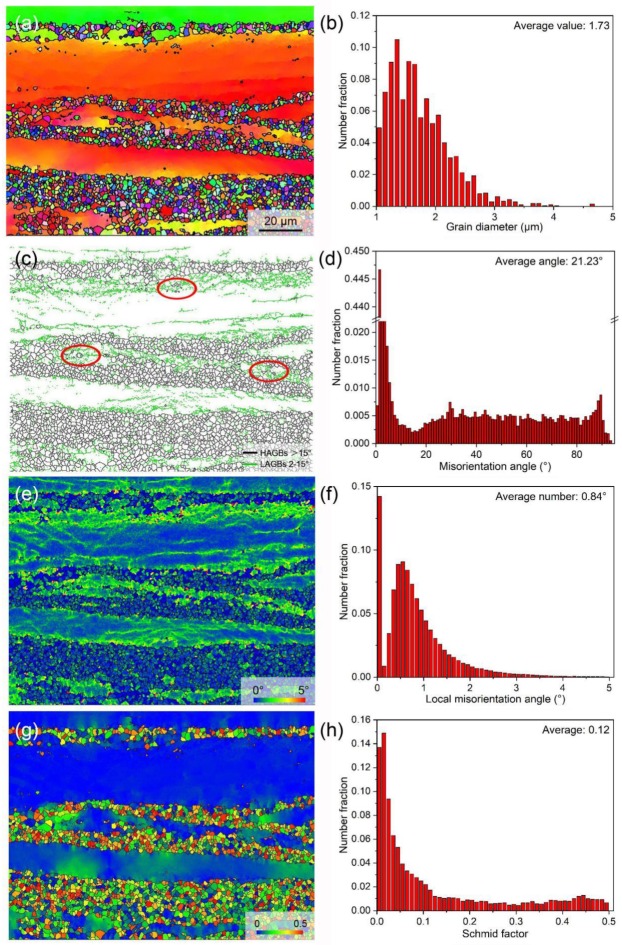
EBSD analysis of the as-extruded sample: (**a**) IPF map with reference direction parallel to TD; (**b**) grain size distribution for DRXed grains; (**c**) GB map and (**d**) misorientation angle distribution; KAM (**e**) map and (**f**) distribution; basal slip Schmid factor (**g**) map and (**h**) distribution along ED.

The EBSD analysis technology was used to examine the information related to the grains of the as-extruded alloy in detail. The inverse pole figure (IPF) map in Figure 5a indicates the typical microstructure composed of both the fine DRXed grains and the coarse elongated un-DRXed grains. The area fractions of DRXed grains and un-DRXed grains are about 32.6% and 67.4%, respectively. The color gradient in a deformed un-DRXed grain suggests that low-angle misorientation still exists in un-DRXed grains. The average size of fine DRXed grains is just about 1.73 μm, as determined by EBSD (Figure 5b). The grain boundary (GB) map shows that numerous low-angle grain boundaries (LAGBs, green lines) are formed in the deformed un-DRXed grains and subdivided them into small substructures (Figure 5c), which is consistent with the color gradient in deformed un-DRXed grains in IPF map (Figure 5a). Moreover, a few DRXed grains can be observed near LAGBs within deformed un-DRXed grains, as marked by red circles in Figure 5c. Therefore, it can be easily inferred that the dynamic recrystallization of this alloy during hot extrusion follows the continuous DRX (C-DRX) mechanism [34,35]. During the hot extrusion process, basal and non-basal dislocations, generated by deformation in the initial grains, develop gradually into LAGBs, and the accumulation of misorientation by the rapid LAGB migration forms new high-angle grain boundaries (HAGBs), resulting in the formation of fine DRXed grains, while some initial grains with low energy storage only develop into the deformed grains with numerous substructures in them, mainly due to insufficient deformation. Figure 5d shows the quantitative analysis of the misorientation angle distributions, revealing the relatively high number fraction of LAGBs. A kernel average misorientation (KAM) map and KAM value distribution are shown in Figure 5e,f. The relatively high misorientations are mainly distributed within the un-DRXed grains and near the DRXed grain group, reflecting the high local strain or dislocation density distribution at these positions, which agrees with the GB map. Figure 5g shows the basal slip Schmid factor map along ED. The elongated un-DRXed grains present negligible basal slip Schmid factors (blue color), while the fine DRXed grains exhibit various basal slip Schmid factors, and the values of most DRXed grains are higher than those of un-DRXed grains. The basal slip Schmid factor distribution with a low average value of 0.12 tends towards lower values (Figure 5h), which is mainly due to the formation of high volume fraction of un-DRXed grains.

**Figure 6:**
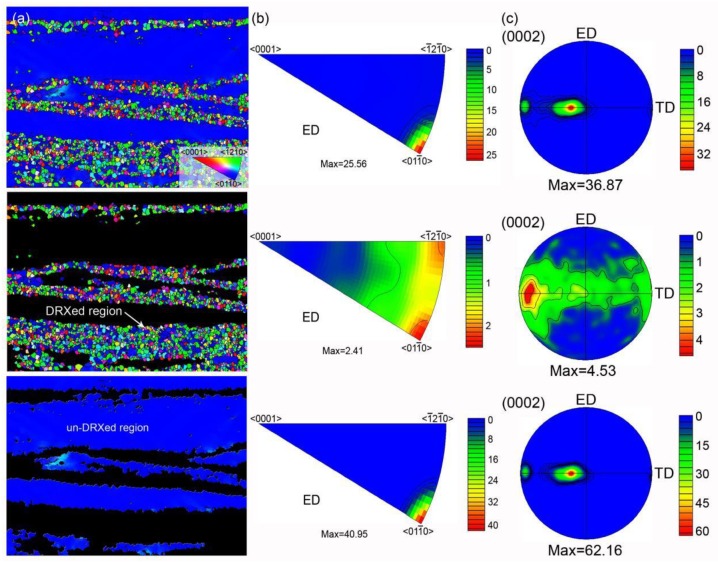
EBSD analysis of whole, DRXed, and un-DRXed regions of the test area from the as-extruded sample: (**a**) IPF maps with the reference direction parallel to ED; (**b**) IPFs referring to the ED; (**c**) basal plane PFs.

Figure 6 shows the IPF maps and IPFs referring to ED, and (0002) pole figures (PFs) of whole region, DRXed region, and un-DRXed region of the as-extruded sample. The IPF maps show that the coarse elongated un-DRXed grains are roughly of a certain orientation, that is, they are blue grains with <01$\bar{1}$0>||ED (i.e., <0001>⊥ED), while the fine DRXed grains are random orientations. Both the IPF and the (0002) PF of whole region reveal that the as-extruded alloy has a very strong basal texture. The IPF and (0002) PF of un-DRXed grains show an extremely similar texture component compared with those of the whole region, except the higher texture intensity, while the IPF and (0002) PF of DRXed grains present very weak basal texture with a large spread of (0002) orientation. The above analysis confirms that the strong basal texture of this as-extruded alloy is mainly caused by the deformed un-DRXed grains. In other words, it also confirms that the DRXed Mg–RE-based extruded alloys generally have weak basal texture.

**Figure 7:**
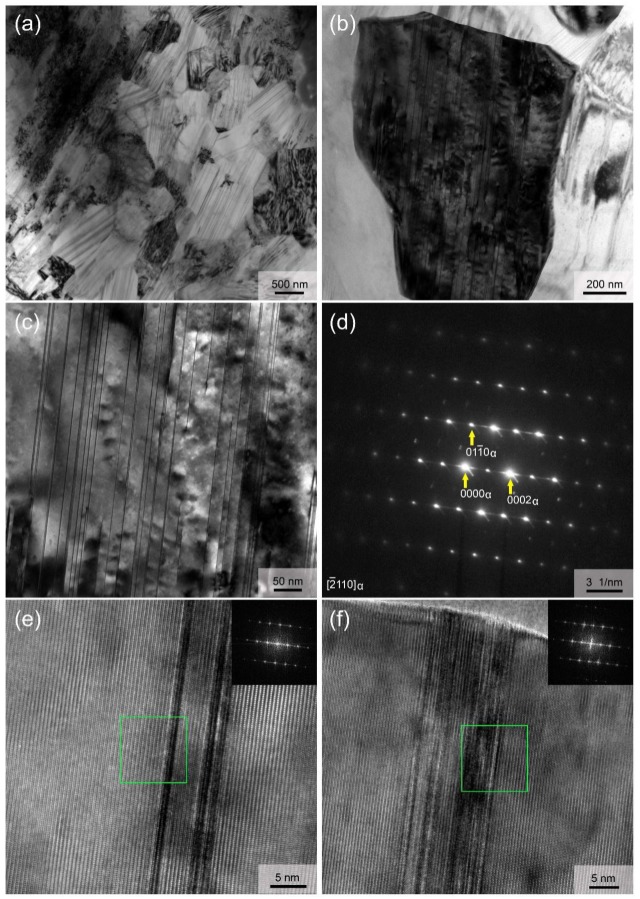
Microstructure of the DRXed region: (**a**–**c**) TEM images; (**d**) SAED pattern; (**e**,**f**) HRTEM images.

TEM was used to observe the internal structure of both DRXed and un-DRXed grains in the as-extruded alloy in detail. Figure 7 mainly shows the TEM analysis of DRXed region. The fine DRXed grains about 1–2 μm in size and fine lamellae within them are formed during hot extrusion. It should be noted that these fine lamellae are only visible at certain viewing angles under TEM. Figure 7b,c provide an example showing the internal structure when only one DRXed grain is concerned. Nanospaced fine lamellae, with spacing ranging from ~5 to ~100 nm, are almost uniformly distributed throughout the whole grain. The corresponding SAED pattern having clear streaks between the diffraction spots along the c-axis (Figure 7d) and the high-resolution TEM (HRTEM) analysis (Figure 7e,f) indicate that these fine lamellae are basal plane SFs [32,33].

**Figure 8:**
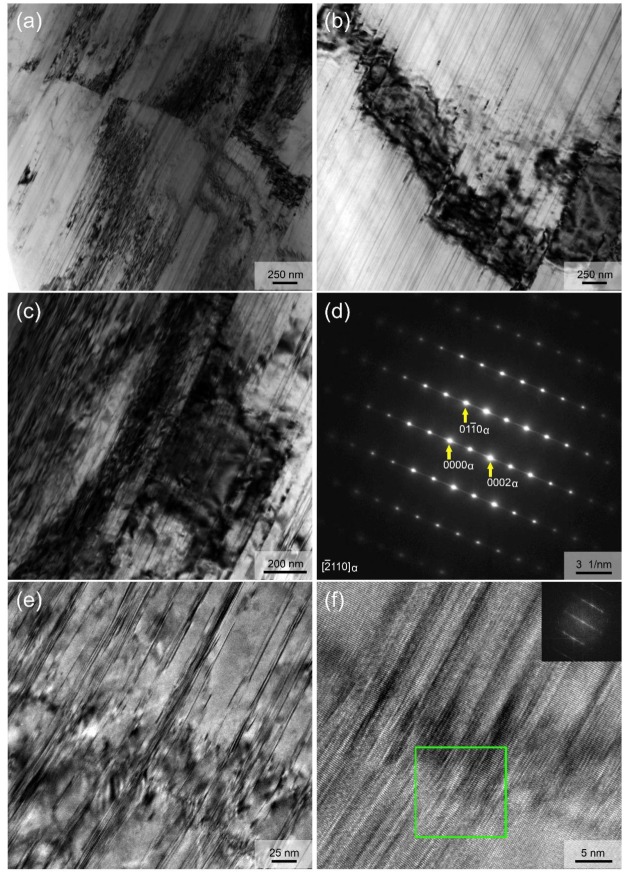
Microstructure of the un-DRXed region: (**a**–**c**,**e**) TEM images; (**d**) SAED pattern; (**f**) HRTEM image.

Figure 8 presents the TEM analysis of un-DRXed region with electron beam parallel to [11$\bar{2}$0]_Mg_. It clearly confirms that the nanospaced lamellae with spacing similar to that of SFs (~5–100 nm) in DRXed grains are also formed in the whole un-DRXed grains during hot extrusion. Moreover, it is noted that these lamellae are not straight, and microstrains and even kinks are formed on the lamellae. The low-angle kinking of SFs corresponds to the LAGB within the deformed un-DRXed grains. The corresponding SAED pattern, having very clear streaks between the diffraction spots along c-axis in Figure 8b, confirms that these fine lamellae are also basal plane SFs [32,33].

**Figure 9:**
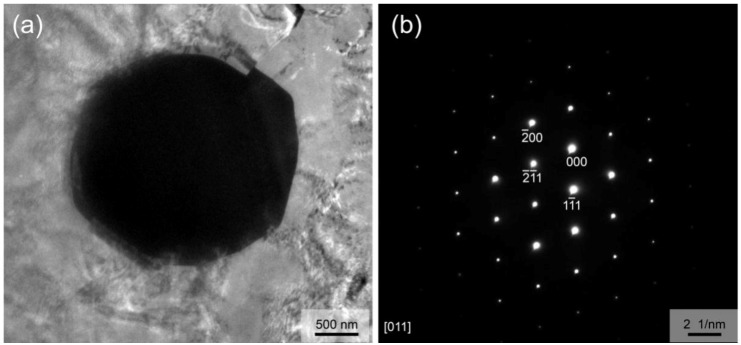
W phase particle in the as-extruded alloy: (**a**) TEM image; (**b**) SAED pattern.

In addition, careful TEM observation also reveals very few particles in the Mg matrix. The particle is an irregular polygon with a size of about 2 μm, as shown in Figure 9a. The corresponding SAED pattern (Figure 9b) confirms that it is W phase. It is considered that these few particles are formed by the crushing of the residual coarse W phase compounds in solid solution-treated alloy during the extrusion process. Considering its trace amounts, it has little effect on the properties of the as-extruded alloy. No LPSO phases were found under TEM observation.

On the whole, we have prepared a new extruded Mg–4Er–2Y–3Zn–0.4Mn (wt %) alloy bar with a special microstructure different from that of previously reported deformed Mg alloys, as reported for the first time. The main novel microscopic feature of this alloy is profuse nanospaced fine basal plane SFs throughout both the whole fine DRXed and deformed un-DRXed grains. The formation of basal plane SFs and their solute segregation have been confirmed and reported in Mg–RE–Zn alloys such as as-cast Mg–Y–Zn alloy [36]. However, these reported SFs usually coexist with plate-shaped LPSO phase in Mg–RE–Zn alloys [36,37], which cannot truly reflect their strengthening effect. Such high number-density SFs strengthened the bimodal microstructure of hot-extruded alloy, and its strengthening effect has not yet been reported. In this study, the formation of this novel microstructure is related to the combination effect of appropriate alloy composition and preparation processing parameters (i.e., solid solution treatment and extrusion process). Selecting a suitable RE (Er and Y) and Zn can lower stacking fault energy and stabilize SFs or LPSO structure [32,38,39] in Mg alloys, while controlling the appropriate processing parameters can ensure the formation of numerous fine SFs, rather than plate-shaped LPSO phase.

### 3.4. Mechanical Properties of the Studied Alloy

**Figure 10:**
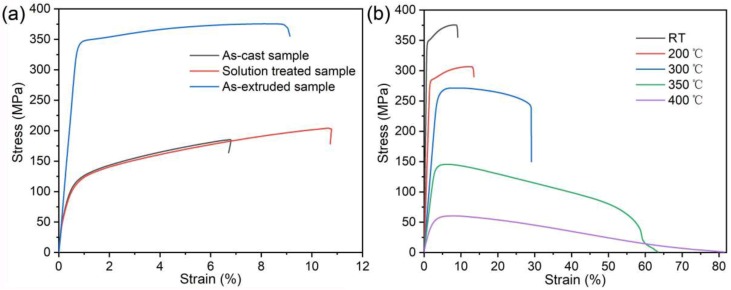
Typical tensile stress–strain curves for (**a**) the studied alloy in different states tested at RT and (**b**) the as-extruded alloy tested at different temperatures.

Figure 10a shows the typical RT tensile stress–strain curves of the studied alloy in as-cast, solid solution-treated, and as-extruded states, and Table 1 lists their ultimate tensile strength (UTS), yield strength (YS), and elongation to failure (El). In the as-cast state, the strength and plasticity of the alloy are low because of the large Mg grain size, and the coarse W and LPSO phases distributed along the dendrite boundaries, whose properties are similar to those of common Mg alloys. After solid solution treatment, most of the secondary phases were dissolved into the matrix, resulting in a slight increase in strength and obvious increase in plasticity, while the strength of the solid solution-treated alloy was dramatically improved after extrusion at 350 °C. Figure 10b shows the typical tensile stress–strain curves of as-extruded alloy tested from RT to 400 °C, and the average tensile properties including UTS, YS, and El are listed in Table 2. By comparing the strength of this new alloy with those of previously reported Mg–RE-based alloys (Table 3 [19,40,41,42,43,44,45]), we find that the newly designed extruded alloy exhibits a superior high-temperature strength at 300 °C compared to the high-temperature Mg alloys containing comparative or even higher RE content. The as-extruded alloy exhibits excellent tensile properties between RT and 300 °C, and the strength was slowly reduced within 300 °C. Both the UTS (375 MPa) and YS (341 MPa) at RT are above 300 MPa. When the test temperature reaches 300 °C, the UTS (271 MPa) and YS (255 MPa) can still remain above 250 MPa. However, when the temperature reached up to 350 °C, the UTS (145 MPa) or YS (131 MPa) decreased sharply, though they were still higher than most of those for reported Mg alloys [2].

When tensile tests are carried out at elevated temperatures, the deformation of Mg alloy should be related not only to the basal dislocation slip, but also non-basal dislocation slips (prismatic slip and pyramidal slip) due to the decrease of critical resolved shear stress (CRSS) with increasing temperature, as well as the grain boundary sliding and lattice rotation [46,47,48,49]. Based on the detailed microstructure observation for the new as-extruded alloy, we deduce that the superior high temperature strength within 300 °C is mainly related to the following factors. First and foremost, the formation of numerous nanospaced basal plane SFs with large aspect ratio in both DRXed and un-DRXed grains should be mainly responsible for the excellent high-temperature strength of the new alloy, since it is the significant difference between the microstructure of the newly designed alloy and those of the reported Mg–RE alloys. The solute-segregated SFs have a coherent interface with the α-Mg matrix and high thermal stability [32,36,38]. On the one hand, these nanospaced basal plane SFs provide effective barriers against dislocation slips, especially non-basal dislocation slips at elevated temperatures. It is considered that the high number-density basal plane SFs contribute much more to the strength than the plate-like basal plane LPSO phase, due to the more pinning [36]. On the other hand, the nanospaced SFs with extremely large aspect ratio can behave as a skeleton for both DRXed and un-DRXed grains to stabilize grains and hinder the grain boundary sliding and lattice rotation, and thus contribute to the superior high-temperature strength. Second, the fine DRXed grains about 2 μm in size contribute to the high YS through grain boundary strengthening according to the Hall–Petch relationship. Third, the deformed un-DRXed grains have very low basal slip Schmid factors along their ED due to their strong basal texture, and thereby sufficiently enhance the YS by providing a hard orientation for the basal dislocation slip. Moreover, the substructures (numerous LAGBs and local strains) within the un-DRXed grains can also serve as barriers for dislocation motion, leading to reinforcement during tensile testing.

## 4. Conclusions

In the present work, a new Mg–4Er–2Y–3Zn–0.4Mn (wt %) alloy was designed and fabricated via semi-continuous casting, solid solution treatment, and hot extrusion. The microstructure in different states and tensile properties at RT and elevated temperatures was investigated. Some main conclusions are listed as follows.

(1) The mixed phases/structures of fcc W phase, 18R-LPSO phase, 14H-LPSO phase and basal plane SFs were formed in the as-cast alloy, mainly based on the alloy component of (Er + Y)/Zn = 1:1 and Er/Y = 1:1 (at %). Heat treatment at 530 °C for 10 h was effective in dissolving the secondary phases into the Mg matrix.
(2) High number-density nanospaced basal plane SFs were formed throughout both the DRXed and un-DRXed grains during hot extrusion at 350 °C for the solid solution-treated alloy. This special feature is the combined effect of appropriate alloy composition and preparation process parameters.
(3) The as-extruded alloy showed superior tensile properties from RT to 300 °C. The UTS and YS could be maintained above 300 MPa at RT, and above 250 MPa even at 300 °C. The excellent elevated-temperature strength is mainly related to the formation of (a) nanospaced basal plane SFs with a large aspect ratio throughout the whole Mg matrix, (b) fine DRXed grains with ~2 μm in size, and (c) strongly basal-textured un-DRXed with numerous substructures.

## Figures and Tables

**Table 1:** Tensile properties of the studied alloy in different states tested at RT.

States	UTS (MPa)	YS (MPa)	El (%)
As-cast	185.3 ± 2.1	89.1 ± 2.9	6.8 ± 0.8
Solution-treated	204.0 ± 2.7	86.8 ± 2.6	10.7 ± 1.0
As-extruded	375.5 ± 4.3	341.4 ± 4.3	9.1 ± 1.8

**Table 2:** Tensile properties of the as-extruded alloy tested at different temperatures.

Temperature (°C)	UTS (MPa)	YS (MPa)	El (%)
25	375.5 ± 4.3	341.4 ± 4.3	9.1 ± 1.8
200	306.6 ± 5.4	276.7 ± 5.7	13.5 ± 1.9
300	271.3 ± 7.4	255.7 ± 4.8	29.1 ± 5.8
350	145.5 ± 2.1	131.5 ± 3.4	63.4 ± 3.7
400	60.5 ± 1.6	36.6 ± 2.7	81.4 ± 3.1

**Table 3:** Tensile property comparison of the studied alloy and the reported high-temperature Mg–RE-based alloys.

Alloys (wt %)	Temperature (°C)	UTS (MPa)	YS (MPa)	El (%)	Refs.
Mg–8Gd–2Y–1Nd–0.3Zn–0.6Zr	RT	271	143	18.7	[40]
	300	188	121	44.0	
Mg–9Gd–4Y–0.6Zr	RT	330	300	13.0	[41]
	300	190	150	28.0	
Mg–11Gd–4.5Y–1Nd–1.5Zn–0.5Zr	RT	377	327	3.8	[42]
	300	193	172	54.4	
Mg–12Gd–3Y–0.5Zr	RT	360	245	2.3	[43]
	300	200	176	18.5	
Mg–9RY–4Zn	RT	265	353	9.7	[44]
	300	204	247	29.2	
Mg–8Gd–3Yb–1.2Zn–0.5Zr	RT	425	413	5.5	[19]
	300	204	191	25.5	
Mg–7Y–4Gd–1.5Zn–0.4Zr	RT	331	228	7.3	[45]
	300	230	123	40.0	
Mg–4Er–2Y–3Zn–0.4Mn	RT	376	341	9.1	This work
	300	271	256	29.1	
